# Processing Differences Between Person and Number: A Theoretical Interpretation

**DOI:** 10.3389/fpsyg.2019.00211

**Published:** 2019-02-14

**Authors:** Peter Ackema, Ad Neeleman

**Affiliations:** ^1^The University of Edinburgh, Edinburgh, United Kingdom; ^2^University College London, London, United Kingdom

**Keywords:** person, number, agreement, processing, features

## Abstract

The literature on processing of person and number agreement contains some apparently contradictory results. On the one hand, some ERP studies do not find a qualitative difference between person and number when an agreeing verb does not match the features of its subject, the controller of the agreement relation (Silva-Pereyra and Carreiras, 2007; Zawiszewski et al., 2016). On the other hand, an ERP study reported in Mancini et al. (2011b) did find a qualitative difference between agreement violations in person and agreement violations in number, a result further corroborated by an fMRI study reported in Mancini et al. (2017). At the same time, there is also a trend on which the literature appears to agree: on the whole the response to agreement violations in person is stronger than the response to number agreement violations. In this paper we argue that the constellation of reported results can be accounted for by adopting a theory of person and number features that has the following two core properties: (i) pronouns are specified for both person and number, but regular NPs are specified for number only and do not carry any person specification; (ii) all of first, second and third person are characterized by one or more person features, whereas, in contrast, one of the numbers (singular) corresponds to the absence of number features.

## Introduction

The literature on processing of person and number agreement contains some apparently contradictory results. On the one hand, some ERP studies do not find a qualitative difference between person and number when an agreeing verb does not match the features of its subject, the controller of the agreement relation (see Silva-Pereyra and Carreiras, 2007; Zawiszewski et al., 2016). On the other hand, an ERP study reported in Mancini et al. (2011a) did find a qualitative difference between agreement violations in person and agreement violations in number, a result further corroborated by an fMRI study reported in Mancini et al. (2017). There is nonetheless also a trend on which the literature appears to agree: on the whole the response to agreement violations in person is stronger than the response to number agreement violations.

The qualitative differences involve both the neuroanatomical and the electrophysiological level. Mancini et al. (2011a: 64) find that “while number agreement violations produced a left-anterior negativity followed by a P600 with a posterior distribution, the negativity elicited by person anomalies had a centro-posterior maximum and was followed by a P600 effect that was frontally distributed in the early phase and posteriorly distributed in the late phase.” One conclusion from Mancini et al.’s (2017) fMRI study is that “while the posterior portion of the (left middle temporal gyrus) is sensitive to both Person and Number Violations, the anterior portion of this region shows selective response for Person Violations” (p. 140).

In contrast, both Silva-Pereyra and Carreiras (2007) and Zawiszewski et al. (2016) explicitly note that they found no qualitative difference in the processing of person agreement violations versus number agreement violations. Both studies do find a quantitative effect. Zawiszewski et al. (2016) note that both person violations and person+number violations elicited larger P600 effects than number violations. Silva-Pereyra and Carreiras (2007) note that the P600 effect induced by a person+number violation is larger than either the effect of a person violation or the effect of a number violation; they did not find a significant difference between the latter two. The existence of a quantitative difference between person and number violations is further confirmed by the study by Mancini et al. (2017), who find a greater response for person compared to number in the region that is sensitive to both (the posterior portion of the left middle temporal gyrus, see above).

We will argue that the apparently contradictory findings can at least partly be understood in terms of the theory of phi-features developed in Ackema and Neeleman (2013, 2018). Two hypotheses play a crucial role in the account. First, pronouns are specified for both person and number, but regular Noun Phrases (which, following the theoretical literature, we will term R-expressions) are specified for number only and do not carry any person features. Second, all of first, second, and third person are characterized by one or more person features. By contrast, one of the numbers, namely singular, corresponds to the absence of number features. Only plurals (and other numbers, such as dual, trial, and paucal) carry one or more number features.

The first hypothesis bears on the contradictory findings described above, because some of the experiments use pronominal subjects as the controller of agreement, while others use R-expressions. The second hypothesis provides a handle on the quantitative difference between the effects of person versus number violations.

The paper is structured as follows. First, we will provide an outline of the theory of person and number features developed in our earlier work (see section “A Theory of Person and Number Features”). Then we will explain how this theory can inform a model of error detection and repair of agreement violations (see section “Detecting and Repairing Agreement Violations”). We will assess how this model fits the reported data in the Section “Accounting for Processing Differences Between Person and Number Violations.” In the final section we mention a further possible test that could be used to assess our model.

## A Theory of Person and Number Features

### The Feature Make-Up of Pronouns

In Ackema and Neeleman (2013, 2018), we propose that there are two privative person features, dubbed PROX (for “proximate”) and DIST (for “distal”). We interpret these features as functions, following insights in Harbour (2011, 2016). Both operate on an input set to deliver a subset as output.

The initial input set for the person system represents all potential referents in a given context (S*_i_*_+_*_u_*_+_*_o_* in (1)). This set has a fixed structure. It contains a subset S*_i_*_+_*_u_*, which itself contains a subset S*_i_*. S*_i_* has the speaker (*i*) as an obligatory member; its other members, if there are any, are associates of the speaker and/or further individuals identified as speaker. S*_i_*_+_*_u_* has one addressee (*u*) as an obligatory member, in addition to all members of S*_i_*; its other members, if there are any, are associates of the addressee and/or further individuals addressed by the speaker. S*_i_*_+_*_u_*_+_*_o_* contains all members of S*_i_*_+_*_u_*; its remaining members, if there are any, are neither associates of the speaker nor of the addressee.


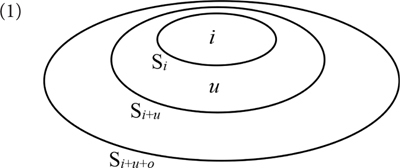


We assume that the input set S*_i_*_+_*_u_*_+_*_o_* is introduced by a category N_Π_, which by definition forms the lexical core of a pronominal expression.

The feature PROX introduces a function that operates on an input set and discards its outermost “layer.” Applied to S*_i_*_+_*_u_*_+_*_o_* it delivers S*_i_*_+_*_u_*. By contrast, DIST introduces a function that *selects* the outermost layer of its input set. Applied to S*_i_*_+_*_u_*_+_*_o_* it delivers S*_i_*_+_*_u_*_+_*_o_* - S*_i_*_+_*_u_*.

This idea can be implemented as follows. Suppose that the various sets in (1) are ordered such that S*_i_* is the predecessor of S*_i_*_+_*_u_*, while S*_i_*_+_*_u_* is the predecessor of S*_i_*_+_*_u_*_+_*_o_* (we will abbreviate “predecessor” as Pred):

(2) a. Pred(S*_i+u_*) = S*_i_*
  b. Pred(S*_i_*_+_*_u_*_+_*_o_*) = S*_i+u_*

If so, characterization of PROX and DIST is simple. The definitions in (3) have the desired effect that PROX discards, while DIST selects, those elements that are part of the outermost layer of the input set:

(3) a. PROX(S) = Pred(S)
  b. DIST(S) = S - Pred(S)

We now consider how first, second and third person readings are derived, starting with the singular. The specification of the third person singular is straightforward: it should be DIST, as this feature will derive S*_i_*_+_*_u_*_+_*_o_* - S*_i_*_+_*_u_*, a set that excludes the speaker and any addressees.

A second person singular reading can be generated by applying both PROX and DIST. Notice that there is only one order of application that yields an interpretation. If PROX is applied first, S*_i_*_+_*_u_* is selected, a set containing the speaker (and any of their associates) and individuals that the speaker addresses (and any of their associates). Applying DIST to this set removes S*_i_*, leaving only addressees (and any associates) as potential members – the required result [see (4)]. In the singular, this will yield a pronoun that refers to exactly one addressee.


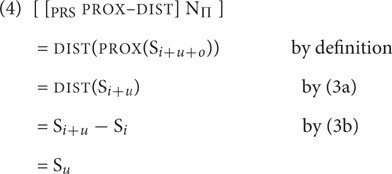


The opposite order of function application is not coherent. DIST applied to S*_i_*_+_*_u_*_+_*_o_* yields S*_i_*_+_*_u_*_+_*_o_* - S*_i_*_+_*_u_* (a set that includes neither the speaker, nor any addressees). But this set is not layered [that is, Pred(S) is not defined for this set]. Therefore, PROX cannot apply to it.

Consider finally the first person. Notice that in the singular just applying PROX to S*_i_*_+_*_u_*_+_*_o_* will not do. This is because the output it delivers, S*_i_*_+_*_u_*, is a set with two obligatory members: the speaker and an addressee. Such a set obviously cannot be construed as singular^1^. Therefore, at least in the singular, a first person reading requires that PROX is applied to the output of PROX. As PROX discards the outermost layer of its input set, this will deliver S*_i_*, a set whose only obligatory member is the speaker and which therefore permits a singular interpretation:


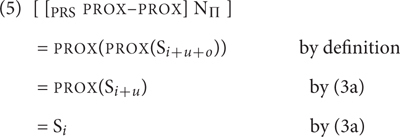


Note that in this person system all persons have one or more features^2^. This contrasts with the classical idea that third person corresponds to the absence of person information (see Benveniste, 1966). There are several previous theories in which the third person is characterized by a feature specification, among them Nevins (2007) and Harbour (2016). Evidence for this hypothesis is intricate, and cannot be reviewed here. It is based on a range of phenomena, including patterns of syncretism in pronominal and verbal agreement paradigms (see Harbour, 2016; Ackema and Neeleman, 2018), dissimilation phenomena in clitic clusters such as Spanish “spurious *se*” (Perlmutter, 1971; Grimshaw, 1997; Nevins, 2007) and person clashes in situations where double agreement has a single morphological reflex (Nevins, 2011; Ackema and Neeleman, 2018).

By contrast, in the number system, there is good evidence that, while there are features for numbers such as plural, dual, and trial, the singular corresponds to the absence of any number features. Evidence for the unmarked status of the singular includes Greenberg (1963: 94) observation that “there is no language in which the plural does not have some non-zero allomorph whereas there are languages in which the singular is expressed only by zero.” Moreover, plural is both a target for morphological impoverishment rules and a context that triggers such rules. This behavior is typical of marked features (see Aalberse and Don, 2009, 2011; Nevins, 2011). Singular does not behave in the same way: it is neither a target nor a context for impoverishment.

We thus arrive at the following inventory of pronominal forms^3^:


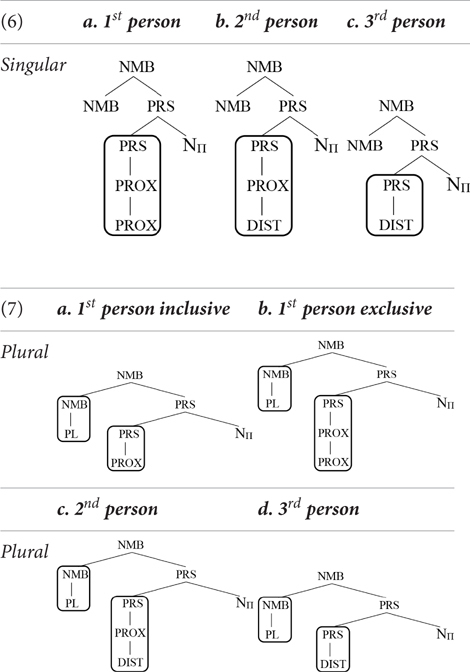


Notice that in the system just outlined, the first person does not form a natural class with the third person to the exclusion of the second person. This fits well with the results of a large-scale study reported in Harbour (2016). Harbour looked at which systematic patterns of syncretism are attested cross-linguistically, where a systematic pattern of syncretism is one that is found in all paradigms of a given language. He reports that no language has a systematic syncretism for first and third person, whereas there are languages that have a systematic syncretism for first and second person, as well as languages that have a systematic syncretism for second and third person. On the assumption that the distribution of systematic syncretisms reflects the underlying distribution of features, this shows that no feature is shared uniquely by first and third person (“uniquely” meaning to the exclusion of second person).

### R-Expressions Do Not Have Person

Even though third person pronouns have a person specification in the system outlined above, Ackema and Neeleman (2018) argue that R-expressions cannot carry person features^4^. They differ from pronouns in not being headed by N_Π_. This means that they do not deliver S*_i_*_+_*_u_*_+_*_o_* to any person features that the R-expression might contain, with the result that these features would be uninterpretable. The evidence that R-expressions do not carry any person information includes the following.

For a start, there are no first or second person R-expressions^5^. A first-person R-expression, for instance, would refer to the speaker and would obligatorily trigger first person agreement. It is certainly possible to use an R-expression to refer to the speaker or addressee (see for instance Collins and Postal, 2012 discussion of what they term “imposters”). However, this is never accompanied by obligatory first or second person agreement. Thus, the English examples in (8) are possible in certain registers, with the subject referring to the speaker. Nonetheless, these R-expressions cannot license first person agreement, let alone that they require it:

(8) a. The present author thinks/^∗^think that this is not justifiable.
  b. Yours Truly has/^∗^have been awarded a Knighthood.

Further evidence that R-expressions like those in (8) are not specified as first person comes from the observation that in discourse they can easily be used ironically to refer to the addressee, as well as the speaker:

(9) A: Yours Truly has been awarded a Knighthood. (*Yours Truly* = speaker)
  B: Well, then Yours Truly must be absolutely thrilled. (*Yours Truly* = addressee)

Crucially, the equivalent is not possible with pronouns, showing that these *are* specified for person. The following is impossible, for instance (no matter how ironic B’s reply is intended to be):

(10) A: I have been awarded a Knighthood. (*I* = speaker)
  B: #Well, then I must be absolutely delighted.(*I* = addressee)

Similar observations can be made for R-expressions that refer to the addressee.

Our proposal implies that R-expressions cannot carry a third person feature either. At first sight, this seems unlikely, given that R-expressions trigger what appears to be third person agreement. However, this is not a particularly compelling argument, because third person “agreement” also shows up in the absence of any possible controller for it: it can function as so-called default agreement. There are a number of languages in which finite clauses without a subject are allowed. In such clauses, the finite verb systematically shows up in its third person form^6^. While we cannot go into this here, it follows from the person system outlined above that a third person feature specification is the only one that need not be interpreted, and therefore the only one allowed on a verb in the absence of a nominal controller. If R-expressions indeed do not have person features, it follows that they should trigger default third person agreement.

There is evidence that R-expressions differ from third person pronouns. Their reference can contain speaker or addressee, as already illustrated in (8) and (9), and as corroborated by the examples in (11). In the latter examples, a first or second person pronoun refers back to an R-expression (underlining is used to indicate intended coreference). By contrast, a third person pronoun cannot be antecedent for a first or second person pronoun, as shown in (12). This follows if third person pronouns are specified as DIST, while R-expressions are not.

(11) a. Anyone who knows the Dutch realizes they no longer wear wooden shoes.
  b. Anyone who knows the Dutch realizes we no longer wear wooden shoes.
  c. Anyone who knows the Dutch realizes you no longer wear wooden shoes.

(12) a. Anyone who knows them realizes they no longer wear wooden shoes.
  b. ^∗^Anyone who knows them realizes we no longer wear wooden shoes.
  c. ^∗^Anyone who knows them realizes you no longer wear wooden shoes.

We conclude that R-expressions do not have person features that determine their reference. They never obligatorily trigger first or second person agreement, and they can be co-referent with any pronoun^7^.

## Detecting and Repairing Agreement Violations

Given the theory outlined in the previous section, let us consider what might happen in processing when the input contains an agreement error. First, of course, the error must be detected. What this means is that the hearer/reader discovers that the features on the agreeing verb are not as expected given the feature specification of the subject. Second, a repair is carried out. We suggest that repair takes the form of deletion of a feature specification, insertion of a feature specification, or both. If both are required, this is a more costly operation than just deletion or insertion.

In principle, this repair can affect either the verb or the subject. There is evidence, at least in the realm of number, that there is variation in this regard. There is a preference to maintain the information on the most recently encountered element, but when repair of the preceding element is impossible it is the most recent element whose feature specification is changed (see Molinaro et al., 2008; Molinaro et al., 2011b). Repair of the subject is impossible, e.g., when the subject is a coordination, which cannot possibly be co-erced into a singular interpretation. For our purposes below, it is the nature of the repair that is crucial, not its location.

Let us first turn our attention to the detection of the agreement error. Here, we expect a qualitative difference between person and number in sentences in which the subject is an R-expression, but not in sentences in which the subject is a pronoun. Consider why. R-expressions are specified for number, but not for person. This implies that if the verb carries incorrect agreement, the type of error is qualitatively different for person and number. For number, the error is a clash: both subject and verb are specified for number, and the verb carries the wrong specification. For person, there is no clash, since the subject does not have person. Because of this, the verb should carry default agreement, which is identical to third person (see section “A Theory of Person and Number Features”). The error, therefore, is that the verb carries a non-default person specification instead^8^. Schematically, the difference between person errors and number errors with subjects that are R-expressions can be represented as follows, where the Greek letters are simply shorthand for a particular feature specification.

(13) NP [NMB: α]… V [NMB: β, PRS: γ]

Note that the specification for number is zero in the singular. This counts as a feature specification, because zero in the context of Num receives an interpretation (“*n* = 1”), distinct from the interpretation of a plural specification. Hence, there is a clash if subject and verb do not agree. However, given that R-expressions lack person altogether, there can be no clash with the person specification of the verb.

The situation is different when the subject is a pronoun, as pronouns have person as well as number. For both types of feature, then, the error will consist of a clash in feature specification:

(14) Pronoun [NMB: α, PRS: β]… V [NMB: γ, PRS: δ]

We are therefore led to expect that in the early stages of processing, when the detection of the agreement error takes place, person and number will behave alike with pronominal subjects, but will show a qualitative difference with subjects that are R-expressions.

Consider next what will happen at the repair stage. For number, there are two possible errors^9^. Either the subject is singular and the verb plural, or the other way around. Above we adopted the hypothesis that singular is a null feature specification. Hence, the two errors can be schematically represented as follows (both with pronouns and with R-expressions):

(15) a. NP/Pronoun [NMB: PL]… V [NMB: __)
  b. NP/Pronoun [NMB: __]… V (NMB: PL)

In both cases, repair is a one-step process. It either consists of insertion of the specification Pl (if the unspecified element is repaired) or deletion of Pl (if the specified element is repaired).

For person, there are more possible errors, simply because there are more person specifications. However, what all these errors have in common is that repair cannot be a one-step process. All persons carry person features (see section “A Theory of Person and Number Features”), and therefore any change in the person specification of the repaired element must involve deletion of one person feature structure, followed by insertion of a different one. For example, if the subject is a first person pronoun, while the verb is third person, the situation is as follows:

(16) Pronoun [PRS: PROX-PROX]… V [PRS: DIST]

If it is the verb that is repaired, [DIST] will be deleted and [PROX-PROX] will be inserted. If it is the subject that is repaired, it is the other way around.

Thus, there is always a quantitative difference in the repair of person errors and number errors. The former is more costly, as it is a two-step process. Since repair obviously takes place after detection, this quantitative difference should present itself later in the process than the qualitative effects related to the detection of agreement errors in the context of R-expressions.

So far, we considered person and number errors separately, but of course the verb can carry a specification that is wrong for both person and number. If so, the repair process will be more costly still, as it must involve three steps: deletion or insertion of a number feature specification, deletion of a person specification, and insertion of a person specification. We may also expect differences in error detection, simply because a double error need not have the same effect as a single one, even disregarding the different nature of the person error with pronominals and R-expressions.

## Accounting for Processing Differences Between Person and Number Violations

The discussion in the previous section gives rise to the following expectations when the verb carries incorrect agreement:

(17) a. The subject is an R-expression: (i) In detection, person behaves differently from number; (ii) In repair, person errors are more costly than number errors.
  b. The subject is a pronoun: (i) In detection, person behaves the same as number; (ii) In repair, person errors are more costly than number errors.

In order to evaluate whether these generalizations hold, we must know what the neurolinguistic footprints might be of detection of an agreement error and its repair.

There is a large amount of literature on the interpretation of different waveforms in ERP studies. Although there does not seem to be a clear consensus on this issue, there are certainly trends. To begin with, a negative waveform between 250 and 500 ms after stimulus onset seems to be associated with unexpected words in the input, including morphosyntactic violations. This negative deflection comes into two or three types. One is the N400. The N400 “is highly correlated (*r* = 0.9) with an offline measure of the eliciting word’s expectancy” (Kutas and Federmeier, 2011: 624). This expectancy is often described in semantic terms, but there are indications that this may be too narrow, at least on the usual linguistic understanding of “semantic.” For example, an N400 effect can be elicited by non-linguistic actions, such as cutting bread with a saw (Proverbio and Riva, 2009; Kutas and Federmeier, 2011). Furthermore, it can be elicited by words that are unexpected in form, but not in semantics. Thus, it is triggered by the form *an* for the indefinite article in English if, in the current context, the noun that is expected to follow starts with a consonant (DeLong et al., 2005). Indeed, for what counts as unexpected, simple word frequency appears relevant (Van Petten and Kutas, 1990). In short, although the N400 does not appear to index semantic anomaly exclusively, or even linguistic anomaly, in the context of language it seems correlated with detecting unexpected words in the input, including certain morpho-syntactic violations (Osterhout, 1997: 497; Tanner and Van Hell, 2014: 298).

There are other types of early negative deflection. In particular, Left Anterior Negativity (LAN) and Anterior Negativity (AN) are plausibly elicited specifically by morpho-syntactic errors, including verb agreement errors as well as case marking errors (Münte et al., 1993, 1997; Friederici et al., 1996; Gunter et al., 1997; Coulson et al., 1998; Friederici and Frisch, 2000).

The size of the LAN effect appears to be partly determined by the morpho-syntax of a language. In particular, it has been observed that it increases the more important agreement is for the parsing of grammatical dependencies (Friederici, 2011: 1381 and references cited there). Agreement is important for detecting the subject of a clause if the agreement paradigm is morphologically rich and therefore reliably indexes the subject’s interpretation. Agreement is also important if the position of the subject in the clause is not fixed, so that word order does not provide a reliable clue as to what the subject is. Conversely, agreement is less important for detecting the subject if word order is strict, or if the morphological verbal agreement paradigm is poor (i.e., contains a lot of syncretisms). Nonetheless, there is some evidence that agreement violations induce a LAN effect also in languages with poorer agreement morphology and a relatively fixed word order, such as Dutch and English (see Osterhout and Mobley, 1995; Hagoort and Brown, 2000; see also Molinaro et al., 2011a for an overview).

Overall, it seems reasonable to correlate the detection of an unexpected agreement form of the verb in the input with a negative waveform in the relevant timeframe. This being said, we should acknowledge that there are some studies of agreement errors that do not find an early negativity effect (see Nevins et al., 2007). Our take on this is that there is a one-way implication: early negativity is in indication of error detection, but error detection is not guaranteed to produce early negativity^10^.

A further clear trend in the literature involves the P600, a positive deflection starting around 500 ms after stimulus onset and lasting a few 100 ms. The P600 is said to be triggered by a range of linguistic anomalies or other difficulties, including those associated with syntactic processing. It is, e.g., triggered by complicated syntax (Kaan et al., 2000; Friederici et al., 2002), less preferred syntactic structure (Osterhout et al., 1994; Itzhak et al., 2010), and by syntactic garden-path effects, i.e., syntactic anomalies that result from misanalysis of an ambiguity rather than from ungrammaticality (Osterhout and Holcomb, 1992, 1993; Osterhout et al., 1994; Kaan and Swaab, 2003). It has also been observed with a variety of syntactic violations, see Gouvea et al. (2010: 150). In view of this, one may expect a P600 effect to index the repair of the morpho-syntactic structure that an agreement violation necessitates.

With the above in mind, let us consider whether the reported effects of agreement violations are in line with (17). One relevant ERP study is reported by Silva-Pereyra and Carreiras (2007). They tested Spanish sentences with pronominal subjects that contained agreement violations of the following three types:

(18) a. Pronoun_1PL_… V_1SG_ (number disagreement)
  b. Pronoun_2SG_… V_1SG_ (person disagreement)
  c. Pronoun_2PL_… V_1SG_ (person and number disagreement)

Given the predictions in (17b), we expect there to be no qualitative differences between any of the examples where it concerns error detection. We expect the errors in (18b) and (18c) to give rise to a more costly repair than the one in (18a), as they involve person. In addition, we expect (18c) to be more costly in repair than (18b), as it involves a double violation.

Silva-Pereyra and Carreiras (2007) found that there were indeed no qualitative differences between person and number violations. They describe their findings as follows (where ND = number disagreement, PD = person disagreement, and NPD = disagreement for both person and number): “ND, PD, and NPD all elicited an anterior negativity (AN) and P600 pattern. An AN effect was only found in the NPD with a different topography from the classic LAN effect as it was lateralized to right and central sites. The P600 effect elicited by the NPD condition was larger than the agreement condition and that of ND and PD in the first window 500–700, while the three disagreement conditions elicited larger P600 amplitudes than the agreement condition in the second window 700–900” (p. 201).

These findings meet our expectations relatively well. The fact that no qualitative differences between person and number were found where it concerns early negativity is the result of pronominal subjects being used in the test sentences. The repair involved in the double violation condition is more costly than the repairs in either single violation condition. The only unexpected finding is the lack of a significant difference between number disagreement and person disagreement in the amplitude of the P600 effect.

Next, an ERP study by Zawiszewski et al. (2016) tested Basque sentences with agreement violations schematized in (19). In this study, too, the subject in all test sentences was a pronoun. The pronoun was always second person singular, while the agreement on the verb was varied to create number disagreement, person disagreement, or disagreement for both person and number.

(19) a. Pronoun_2SG_… V_2PL_ (number disagreement)
  b. Pronoun_2SG_… V_1SG_ (person disagreement)
  c. Pronoun_2SG_… V_1PL_ (person and number disagreement)

On the basis of (17b), we again expect no qualitative differences in the effects triggered by the various violations. We do expect a quantitative difference in the repair stage of the process, where the violations that involve person should trigger a larger effect than the number violation. We also expect the double violation to give the largest effect in repair.

These expectations are largely met. Zawiszewski et al. (2016) found that, first, all violation types triggered an N400-P600 pattern. Second, person and person+number violations elicited larger P600 effects than number violations. To be more specific, with regards to the N400, “no differences were found between person and number violations or between person and person+number violations, while number violations elicited a larger negativity over left-posterior sites than person+number violations.” With regards to the P600, “no differences were found between person and person+number violations, while both person and person+number violations elicited a larger P600 than number violations over posterior sites accompanied by a larger negativity over frontocentral sites” (p. 618). Zawiszewski et al. (2016):618) summarize their findings as follows: “Our results revealed qualitatively similar but quantitatively larger ERP signatures for person than for number violations.”

This conclusion supports our main contention: if pronominal subjects are used, no qualitative differences between person and number violations are to be expected. Also, the hypothesis that violations involving person should always give rise to a more costly repair than violations only involving number, and hence to larger P600 effects, is confirmed. We do not have a specific account for the difference between the number violation and the double violation with respect to the amplitude of the N400, nor for the absence of a significant difference in the size of the P600 in the double violation condition and the person disagreement condition. (Note that the latter finding is the opposite of what Silva-Pereyra and Carreiras found).

A third relevant ERP study is the one by Mancini et al. (2011a). They tested Spanish sentences with R-expressions as subject. The test sentences were of the following types:

(20) a. NP_SG_… V_3PL_ (number disagreement)
  b. NP_SG_… V_2SG_ (person disagreement)

Note that we have not labeled the NP subject as being third person, in line with our hypothesis that R-expressions do not have person. The structure in (20b) is therefore not actually a case of disagreement; rather, the verb does not show the expected default third person agreement that is selected when the controller does not have person features. Crucially, this is a different type of error than the one in (20a), where there is a clash between the number specification of the subject (namely ø) and the number specification of the verb; see section “A Theory of Person and Number Features” for more detailed discussion. Hence, as mentioned in (17a), we should find qualitative differences between the sentences with a person violation and the sentences with a number violation.

Mancini et al. indeed found that the parser is differentially sensitive to the two features. “While number agreement violations produced a left-anterior negativity followed by a P600 with a posterior distribution, the negativity elicited by person anomalies had a centro-posterior maximum and was followed by a P600 effect that was frontally distributed in the early phase and posteriorly distributed in the late phase” (Mancini et al., 2011a: 64).

In addition, “both anomalies produce a P600 effect that has its maximum in posterior sites. Differences between number and person emerged in terms of the amplitude of this effect, which appears to be larger for the person mismatch” (Mancini et al., 2011a: 73). The latter observation confirms the second prediction in (17a), namely that repair of person violations is more costly than repair of number violations regardless of the nature of the subject.

A final relevant study, by Mancini et al. (2017), uses fMRI, rather than ERP, as its investigative technique. This implies that there is not enough temporal resolution to distinguish the detection and repair stages of the processing of sentences with agreement errors. However, fMRI can of course identify qualitative and quantitative differences in the parsing process, and so it does provide an opportunity to test the generalizations in (17) provided their temporal dimension, and hence the distinct reference to the detection and repair stages of the process, are removed. If we do this, the generalizations are as follows:

(21) a. The subject is an R-expression: Person behaves qualitatively differently from number and will have a quantitatively larger effect.
  b. The subject is a pronoun: There are no qualitative differences between person and number, but person will have a quantitatively larger effect.

Mancini et al. tested Spanish sentences with an R-expression as subject, containing an agreement error of one of the following two types:

(22) a. NP_SG_… V_3PL_ (number disagreement)
  b. NP_SG_… V_2SG_ (person disagreement)

Since the subjects are R-expressions, we expect the behavior in (21a). This is in line with what Mancini et al. found: “The direct contrast between Person and Number Violations permitted the uncovering of both quantitative and qualitative differences” (p. 147). More specifically, “A greater response for person compared to number was found in the left middle temporal gyrus (LMTG). However, critically, a posterior-to-anterior functional gradient emerged within this region. While the posterior portion of the LMTG was sensitive to both Person and Number Violations, the anterior portion of this region showed selective response for Person Violations” (p. 140).

To the best of our knowledge, there is no similar fMRI study that compares person and number agreement violations, but uses pronominal subjects instead. For now, then, the prediction in (21b) is left untested.

In sum, the studies that use pronominal subjects to explore agreement errors do not find qualitative differences between person and number, while the studies that use R-expressions as subject do find such differences. This is accounted for by a theory in which pronouns have a person specification, but R-expressions do not.

Studies differ in where they find significant quantitative differences between number violations, person violations and double violations. However, all significant differences that were found follow a hierarchy number < person < number+person. This is in line with the idea that repair of person violations is more costly than repair of number violations, though perhaps the difference in cost is relatively small.

## A Possible Further Experiment

Our proposal can be tested further, as one crucial data set currently remains unexplored. This involves third person pronouns. In traditional grammar, such pronouns are treated on a par with what we call R-expressions: both are third person. However, on the theory proposed here only the pronouns carry a third person specification; the R-expressions are personless. Hence, the prediction is that there should no contrastive behavior between third person pronouns and first and second person pronouns. The contrast should be between all pronouns on the one hand and R-expressions on the other hand. For example, an agreement violation between a third person singular pronoun and, say, a first person singular verb is of the type in (23a), and therefore involves a clash in person features. An agreement violation between a singular R-expression and a first person singular verb is, as discussed, of the type in (23b), which does not involve such a clash.

(23) a. Pronoun [NMB: __, PRS: DIST]… V [NMB: __, PRS: PROX-PROX]
  b. NP [NMB: __]… V [NMB: __, PRS: PROX-PROX]

Relevant examples with third person pronominal subjects have not been tested, as far as we know. We expect that there are qualitative differences in error detection between the two conditions in (23).

## Conclusion

We hope to have shown that theoretical accounts of agreement can be used to interpret experimental data, and that experimental data can be used to test theoretical accounts. In particular, we have argued that contrasts between the processing of person and number agreement violations may fall out from a specific theory of person and number features according to which (i) R-expressions do not have person, while pronouns do, and (ii) singular is the absence of a number feature, while all persons, including third person, have person features. To the extent that other theoretical accounts of person and number make different predictions, the experimental data can be said to confirm this theory.

## Author Contributions

All authors listed have made a substantial, direct and intellectual contribution to the work, and approved it for publication.

## Conflict of Interest Statement

The authors declare that the research was conducted in the absence of any commercial or financial relationships that could be construed as a potential conflict of interest.

## Footnotes

1 We assume that number in the context of pronouns is a feature that reflects the cardinality of its input set, rather than a feature (like person) that selects a subset from this input set (see Ackema and Neeleman, 2018: chapter 3 for more details). This means that there is no option to interpret a pronoun specified as [PROX] as singular by having it refer to just the *i* or just the *u* that are contained in the output of the person system (S*_i_*_+_*_u_* in this case).

2 In a system with privative features such as this, we may expect pronouns unmarked for person features. These arguably exist but correspond to impersonal rather than personal pronouns (an example of an impersonal pronoun is the English generic pronoun *one* as in *one can see the Eiffel Tower from here*); see for instance Egerland (2003); D’Alessandro (2007), Nevins (2007); Ackema and Neeleman (2018) for discussion.

3 The difference between (7a) and (7b) corresponds to the distinction between first person inclusives and exclusives, a distinction that is morphologically marked in a number of languages. For our present purposes, this distinction is not relevant.

4 The question of whether R-expressions and third person pronouns differ in their specification for person is not often discussed in detail. In many proposals, the two categories are treated on a par in that both or neither are specified for person. There are a few exceptions that make the same cut that we take to be crucial; see in particular Sichel, 2000.

5 Simona Mancini points out that Basque NPs that carry the proximate plural article *–ok* might be a counterexample to this generalization. We do not think so; Trask (2003: 122) notes that in the relevant varieties a phrase like *gizonok* variously means “we men,” “you men,” or “the men here.” This is consistent with an analysis of *–ok* as a regular proximate marker, but not with an analysis as a person marker, as the person appears to vary across these translations. (For some discussion of expressions like *we men*, see Ackema and Neeleman, 2018: 155ff. and 294–295).

6 A related observation can be made for English. There are finite clauses that have a subject, but not one that has phi-features. A case in point are clauses that have a sentential subject. In this context, too, the finite verb carries third person agreement (e.g., *That Mary wants to move to Ireland surprises/^∗^surprise no one*).

7 We should note that this conclusion does not imply that there are no contexts in which R-expressions carry first or second person morphology. This is possible if the morphology in question is agreement morphology, and therefore not interpreted in the R-expression itself. In particular, when an R-expression has a predicative function, it may agree with a first or second person subject. This can be observed, e.g., in Classical Nahuatl (Launey, 2011) and Mohawk (Baker, 1996).

8 In fact, in some pro drop languages plural R-expressions can be combined with a first or second person verb if they refer to a group of people containing the speaker or addressee (a phenomenon known as “unagreement,” see Hurtado, 1985). Pronouns never permit this possibility. There is a variety of analyses available (e.g., Ackema and Neeleman, 2013; Höhn, 2016), but it should be clear that the hypothesis that R-expressions do not have person provides a good basis for an explanation. Unagreement is generally not possible in the singular, which is why the experiments reported below use either pronouns or singular R-expressions as subject. Unagreement gives rise to an ERP profile that is distinct from regular agreement as well as from agreement errors, see Mancini et al. (2011b).

9 That is, possible errors in the context of the experiments discussed. There are more logically possible errors if we include numbers other than singular and plural (such as dual and trial). As far as we know, there is no experimental literature testing examples with other numbers yet, and we will put them to one side.

10 If so, studies that do not find early negativity can also not identify potential differences in the detection of person and number agreement errors.

## References

[B1] Aalberse S. Don J. (2009). Syncretism in Dutch dialects. *Morphology* 19 3–14. 10.1007/s11525-009-9132-y

[B2] Aalberse S. Don J. (2011). Person and number syncretisms in Dutch. *Morphology* 21 327–350. 10.1007/s11525-010-9164-3

[B3] Ackema P. Neeleman A. (2013). Subset controllers in agreement. *Morphology* 23 291–323. 10.1007/s11525-013-9218-4

[B4] Ackema P. Neeleman A. (2018). *Features of Person: From the Inventory of Persons to their Morphological Realization.* Cambridge, MA: MIT Press.

[B5] Baker M. (1996). *The Polysynthesis Parameter.* Oxford: Oxford University Press.

[B6] Benveniste É (1966). *Problèmes de Linguistique Générale.* Paris: Gallimard.

[B7] Collins C. Postal P. (2012). *Imposters: A Study of Pronominal Agreement.* Cambridge, MA: MIT Press 10.7551/mitpress/9780262016889.001.0001

[B8] Coulson S. King J. W. Kutas M. (1998). Expect the unexpected: event-related brain response to morphosyntactic violations. *Lang. Cogn. Process.* 13 21–58. 10.1080/016909698386582

[B9] D’Alessandro R. (2007). *Impersonal Si-Constructions.* Berlin: Mouton de Gruyter 10.1515/9783110207514

[B10] DeLong K. A. Urbach T. P. Kutas M. (2005). Probabilistic word pre-activation during language comprehension inferred from electrical brain activity. *Nat. Neurosci.* 8 1117–1121. 10.1038/nn1504 16007080

[B11] Egerland V. (2003). Impersonal pronouns in Scandinavian and Romance. *Work. Pap. Scand. Syntax* 71 75–102.

[B12] Friederici A. D. (2011). The brain basis of language processing: from structure to function. *Physiol. Rev.* 91 1357–1392. 10.1152/physrev.00006.2011 22013214

[B13] Friederici A. D. Frisch S. (2000). Verb argument structure processing: the role of verb-specific and argument-specific information. *J. Mem. Lang.* 43 476–507. 10.1006/jmla.2000.2709

[B14] Friederici A. D. Hahne A. Mecklinger A. (1996). Temporal structure of syntactic processing: early and late event-related potential effects. *J. Exp. Psychol. Learn. Mem. Cogn.* 22 1219–1248. 10.1037/0278-7393.22.5.1219 8805821

[B15] Friederici A. D. Steinhauer K. Pfeifer E. (2002). Brain signatures of artificial language processing: evidence challenging the critical period hypothesis. *PNAS* 99 529–534. 10.1073/pnas.012611199 11773629 PMC117594

[B16] Gouvea A. C. Phillips C. Kazanina N. Poeppel D. (2010). The linguistic processes underlying the P600. *Lang. Cogn. Process.* 25 149–188. 10.1080/01690960902965951

[B17] Greenberg J. (1963). “Some universals of grammar with particular reference to the order of meaningful elements,” in *Universals of Human Language*, ed. Greenberg J. (Cambridge, MA: MIT Press), 58–90.

[B18] Grimshaw J. (1997). “The best clitic: constraint conflict in morphosyntax,” in *Elements of Grammar*, ed. Haegeman L. (Dordrecht: Kluwer), 169–196.

[B19] Gunter T. C. Stowe L. A. Mulder G. (1997). When syntax meets semantics. *Psychophysiology* 34 660–676. 10.1111/j.1469-8986.1997.tb02142.x 9401421

[B20] Hagoort P. Brown C. M. (2000). ERP effects of listening to speech compared to reading: the P600/SPS to syntactic violations in spoken sentences and rapid serial visual presentation. *Neuropsychologia* 38 1531–1549. 10.1016/S0028-3932(00)00053-1 10906378

[B21] Harbour D. (2011). Valence and atomic number. *Linguist. Inq.* 42 561–594. 10.1162/LING_a_00061

[B22] Harbour D. (2016). *Impossible Persons.* Cambridge, MA: MIT Press 10.7551/mitpress/9780262034739.001.0001

[B23] Höhn G. (2016). Unagreement is an illusion: apparent person mismatches and nominal structure. *Nat. Lang. Linguist. Theory* 34 543–592. 10.1007/s11049-015-9311-y

[B24] Hurtado A. (1985). “The Unagreement Hypothesis,” in *Selected Papers from the Thirteenth Linguistic Symposium on Romance Languages*, eds King L. D. Maley C. A. (Amsterdam: John Benjamins), 187–211. 10.1075/cilt.36.12hur

[B25] Itzhak I. Pauker E. Drury J. E. Baum S. R. Steinhauer K. (2010). Event-related potentials show online influence of lexical biases on prosodic processing. *Neuroreport* 21 8–13. 10.1097/WNR.0b013e328330251d 19884867

[B26] Kaan E. Harris A. Gibson E. Holcomb P. J. (2000). The P600 as an index of syntactic integration difficulty. *Lang. Cogn. Process.* 15 159–201. 10.1080/016909600386084 15722211

[B27] Kaan E. Swaab T. Y. (2003). Repair, revision, and complexity in syntactic analysis: an electrophysiological differentiation. *J. Cogn. Neurosci.* 15 98–110. 10.1162/089892903321107855 12590846

[B28] Kutas M. Federmeier K. D. (2011). Thirty years and counting: finding meaning in the N400 component of the event related brain potential (ERP). *Annu. Rev. Psychol.* 62 621–647. 10.1146/annurev.psych.093008.131123 20809790 PMC4052444

[B29] Launey M. (2011). *An Introduction to Classical Nahuatl.* Cambridge: Cambridge University Press 10.1017/CBO9780511778001

[B30] Mancini S. Molinaro N. Rizzi L. Carreiras M. (2011a). A person is not a number: discourse involvement in subject-verb agreement computation. *Brain Res.* 1410 64–76. 10.1016/j.brainres.2011.06.055 21798519

[B31] Mancini S. Molinaro N. Rizzi L. Carreiras M. (2011b). When persons disagree: an ERP study of unagreement in Spanish. *Psychophysiology* 48 1361–1371. 10.1111/j.1469-8986.2011.01212.x 21517901

[B32] Mancini S. Quiñones I. Molinaro N. Hernandez-Cabrera J. A. Carreiras M. (2017). Disentangling meaning in the brain: left temporal involvement in agreement processing. *Cortex* 86 140–155. 10.1016/j.cortex.2016.11.008 27984788

[B33] Molinaro N. Barber H. A. Carreiras M. (2011a). Grammatical agreement processing in reading: ERP findings and future directions. *Cortex* 47 908–930. 10.1016/j.cortex.2011.02.019 21458791

[B34] Molinaro N. Vespignani F. Zamparelli R. Job R. (2011b). Why “brother and sister” are not just “siblings”: repair processes in agreement computation. *J. Mem. Lang.* 64 211–232. 10.1016/j.jml.2010.12.002

[B35] Molinaro N. Kim A. Vespignani F. Job R. (2008). Anaphoric agreement violation: an ERP analysis of its interpretation. *Cognition* 106 963–974. 10.1016/j.cognition.2007.03.006 17445790

[B36] Münte T. F. Heinze H.-J. Mangun G. R. (1993). Dissociation of brain activity related to syntactic and semantic aspects of language. *J. Cogn. Neurosci.* 5 335–344. 10.1162/jocn.1993.5.3.335 23972221

[B37] Münte T. F. Matzke M. Johannes S. (1997). Brain activity associated with syntactic incongruencies in words and pseudo-words. *J. Cogn. Neurosci.* 9 318–329. 10.1162/jocn.1997.9.3.318 23965010

[B38] Nevins A. (2007). The representation of third person and its consequences for person-case effects. *Nat. Lang. Linguist. Theory* 25 273–313. 10.1007/s11049-006-9017-2

[B39] Nevins A. (2011). Multiple agree with clitics: person complementarity vs. omnivorous number. *Nat. Lang. Linguist. Theory* 29 939–971. 10.1007/s11049-011-9150-4

[B40] Nevins A. Dillon B. Malhotra S. Phillips C. (2007). The role of feature-number and feature-type in processing Hindi verb agreement violations. *Brain Res.* 1164 81–94. 10.1016/j.brainres.2007.05.058 17658491

[B41] Osterhout L. (1997). On the brain response to syntactic anomalies: manipulations of word position and word class reveal individual differences. *Brain Lang.* 59 494–522. 10.1006/brln.1997.1793 9299074

[B42] Osterhout L. Holcomb P. J. (1992). Event-related brain potentials elicited by syntactic anomaly. *J. Mem. Lang.* 31 785–806. 10.1016/0749-596X(92)90039-Z

[B43] Osterhout L. Holcomb P. J. (1993). Event-related potentials and syntactic anomaly: evidence of anomaly detection during the perception of continuous speech. *Lang. Cogn. Process.* 8 413–438. 10.1080/01690969308407584

[B44] Osterhout L. Holcomb P. J. Swinney D. A. (1994). Brain potentials elicited by garden-path sentences: evidence for the application of verb information during parsing. *J. Exp. Psychol. Learn. Mem. Cogn.* 20 786–803. 10.1037/0278-7393.20.4.786 8064247

[B45] Osterhout L. Mobley L. A. (1995). Event-related brain potentials elicited by failure to agree. *J. Mem. Lang.* 34 739–773. 10.1006/jmla.1995.1033

[B46] Perlmutter D. (1971). *Deep and Surface Structure Constraints in Syntax.* New York, NY: Holt, Rinehart, and Winston.

[B47] Proverbio A. M. Riva F. (2009). RP and N400 ERP components reflect semantic violations in visual processing of human actions. *Neurosci. Lett.* 459 142–146. 10.1016/j.neulet.2009.05.012 19427368

[B48] Sichel I. (2000). “Demonstrative pronouns, binding theory, and identity,” in *Proceedings of the 24th Annual Penn Linguistics Colloquium* (Philadelphia, PA: University of Pennsylvania), 247–259.

[B49] Silva-Pereyra J. F. Carreiras M. (2007). An ERP study of agreement features in Spanish. *Brain Res.* 1185 201–211. 10.1016/j.brainres.2007.09.029 17963736

[B50] Tanner D. Van Hell J. G. (2014). ERPs reveal individual differences in morphosyntactic processing. *Neuropsychologia* 56 289–301. 10.1016/j.neuropsychologia.2014.02.002 24530237

[B51] Trask L. (2003). “Morphology,” in *A Grammar of Basque*, eds Hualde J. I. Ortiz de Urbina J. (Berlin: Mouton de Gruyter), 113–362.

[B52] Van Petten C. Kutas M. (1990). Interactions between sentence context and word frequency in event-related brain potentials. *Mem. Cogn.* 18 380–393. 10.3758/BF03197127 2381317

[B53] Zawiszewski A. Santesteban M. Laka I. (2016). Phi-features reloaded: an even-related potential study on person and number agreement processing. *Appl. Psycholinguist.* 37 601–626. 10.1017/S014271641500017X

